# High body mass index is a predictor of left ventricular reverse remodelling in heart failure with reduced ejection fraction

**DOI:** 10.1002/ehf2.12172

**Published:** 2017-07-27

**Authors:** Arthur Cescau, Lucas N.L. Van Aelst, Mathilde Baudet, Alain Cohen Solal, Damien Logeart

**Affiliations:** ^1^ Department of Cardiology Hôpital Lariboisière Paris 75010 France; ^2^ Department of Cardiovascular Sciences KU Leuven Campus Gasthuisberg Leuven 3000 Belgium; ^3^ U942 INSERM Paris France; ^4^ Sorbonne Paris Cité University Paris VII – Denis Diderot Paris France

**Keywords:** Heart failure, Obesity, Echocardiography, Cardiac remodelling

## Abstract

**Aims:**

Structural and functional left ventricular alterations can occur in heart failure (HF), referred to as left ventricular reverse remodelling (LVRR). This study aimed to define novel predictors of LVRR besides well‐known effects of medical and device therapy.

**Methods and results:**

From echographic database, we included 295 patients with both left ventricular ejection fraction (LVEF) ≤45% and indexed left ventricular end‐diastolic diameter ≥33 mm/m^2^ and who had at least two echocardiographic exams with a delay between 3 and 12 months. LVRR was defined as the combination of (i) normalization of LVEF (LVEF ≥50%) or increase in LVEF ≥10% and (ii) a decrease in indexed left ventricular end‐diastolic diameter ≥10%. Clinical follow‐up was also obtained. LVRR occurred in 53 (18%) patients. Patients in the LVRR group were more likely to present with *de novo* HF (75% vs. 42%), had lower LVEF and left ventricular end‐diastolic volumes at index examination, yet a higher body mass index (BMI) than non‐LVRR patients. Obesity was observed in 25% of LVRR patients vs. 14% in others. In multivariate analyses, BMI (per each 1 kg/m^2^ increase) emerged as a predictor of LVRR: odds ratio 1.10 (95% confidence interval 1.02–1.19) after adjustment to other predictors of LVRR. During a mean follow‐up of 37 months, 32% of patients had a major adverse cardiac event; *de novo* HF, age, and LVEF were associated with major adverse cardiac event.

**Conclusions:**

We identified significant relationship between high BMI and LVRR. This intriguing novel finding deserves further study.

## Introduction

Heart failure (HF) is associated with structural and functional changes in the myocardium, referred to as ventricular remodelling. Left ventricular remodelling (LVR) further decreases ventricular performance and is strongly related to adverse outcome.^1^ Left ventricular end‐systolic volume and left ventricular end‐diastolic volume have been proposed as the most reliable parameters of LVR, as these convey both structural and functional information, yet their respective critical to define LVR is debated.^2^ Guideline‐based therapies, both drugs and devices, can reverse the detrimental alterations to the left ventricle, referred to as left ventricular reverse remodelling (LVRR). In contrast with LVR, LVRR has been associated with improved survival.^3^, ^4^, ^5^

In this study, we aimed to unravel new clinical predictors of LVRR and evaluate their impact on HF prognosis, by using a well‐defined population of HF patients with regular clinical and echocardiographic follow‐up.

## Methods

Our echocardiographic database was analysed retrospectively to identify patients who were referred between January 2010 and January 2015 because of HF as well as ischaemic heart disease or dilated cardiomyopathy. Included patients were at least 18 years old and had at least two echocardiographic exams performed with an interval of 3 to 12 months between the two exams. Included patients presented with a left ventricular ejection fraction (LVEF) below 45% and an indexed left ventricular end‐diastolic diameter (LVEDDi) of at least 33 mm/m^2^ at the index echographic exam.

The clinical database was matched to echographic database in order to obtain main clinical characteristics as well as treatments. We excluded patients with acute coronary syndrome as well as pacing, cardioversion, and cardiac surgery that occurred during the 3 months before inclusion as well as between the two echographic exams. Patients with non‐sinus rhythm and severe valvular disease were also excluded.

Echocardiographic measurements were performed by experienced cardiologists on Philips iE33 (Amsterdam, Netherlands) or GE Vivid 9 (Chicago, IL, USA) ultrasound systems.^6^ Weight data were recorded at each examination. LVEF was measured using Simpson method. Authors involved in image acquisition were not involved in further statistical analysis of the data and vice versa. LVRR was defined as the combination of (i) an increase in LVEF of at least 10% or normalization of LVEF (LVEF ≥50%) and (ii) a decrease in LVEDDi of at least 10%.^7^, ^8^ Clinical follow‐up was obtained, and major adverse cardiac event (MACE) was defined as all‐cause death or the need for cardiac transplantation. The investigation conforms with the principles outlined in the Declaration of Helsinki*.*

Continuous variables are expressed as means with standard deviations when normally distributed or as medians with interquartile ranges when not normally distributed. Normality was assessed by the Shapiro–Wilk test. Categorical variables are presented as numbers and percentages. Groups were compared using Student's *t*‐test or non‐parametric alternatives as appropriate. Variables were assessed for their potential to predict LVRR as well as MACE in univariate and multivariate analyses and are presented with their respective odds ratios (OR) and 95% confidence intervals (CI). For multivariate analyses, all predictors were forced into models simultaneously. A *P*‐value of <0.05 was considered statistically significant. Statistical analyses were performed using STATA 14.1 (StataCorp, Texas, USA).

## Results

The mean duration between the two examinations at 6 months (interquartile range 4–11). Changes in body weight were very small. Among 295 included patients LVEF increased by ≥0.10 in 72 and normalizes (LVEF ≥50%) in 25 patients while LVEDDi decreased by ≥10% in 55 patients. Combining these two parameters, LVRR occurred in 53 patients (18%). On the other hand, LVEF decreased by ≥0.10 in 16 patients and LVEDDi increases by ≥10% in 25 patients. Main characteristics are shown according to the occurrence or not of LVRR in *Table* 1. In multivariate analysis including age, *de novo* HF, treatment, cardiac resynchronization therapy, body mass index (BMI), LVEDDi, and LVEF, only presentation with *de novo* HF, BMI (per 1 kg/m^2^ increase), and LVEDDi (per 1 mm/m^2^ increase) were associated with LVRR: OR 4.22 (95%CI 1.81–9.80), OR 1.10 (95%CI 1.02–1.19), and OR 0.98 (95%CI 0.99), respectively. Obesity *per se* was at limit of significance (OR 1.96, 95%CI 0.95–4.04).

**Table 1:** Clinical characteristics of patients, categorized according to left ventricular reverse remodeling status during follow‐up

	Total cohort *n* = 295	LVRR absent *n* = 242	LVRR present *n* = 53
Age (y)	66 [57–74]	66 [58–75]	63 [55–73]
Male/female (%)	83/17	85/15	74/26
Alcohol use: no/yes/missing (%)	82/17/1	83/17/0	79/19/2
Current smoker	167 (57%)	135 (56%)	32 (60%)
Diabetes	90 (31%)	74 (31%)	16 (30%)
Arterial hypertension	181 (61%)	147 (61%)	34 (64%)
Ischaemic CMP	142 (48%)	119 (49%)	23 (43%)
Dilated CMP	105 (36%)	87 (36%)	18 (34%)
*De novo* HF*	142 (48%)	102 (42%)	40 (75%)
ACE‐I/ARB	288 (98%)	236 (98%)	52 (98%)
Beta blocker	263 (89%)	216 (89%)	47 (89%)
Aldosterone antagonist	155 (53%)	130 (54%)	25 (47%)
Loop diuretic*	259 (88%)	217 (90%)	42 (79%)
Cardiac rehabilitation	117 (40%)	94 (39%)	23 (43%)
ICD*	78 (26%)	74 (31%)	4 (8%)
CRT*	40 (14%)	39 (16%)	1 (2%)
Obesity	47 (16%)	34 (14%)	13 (25%)
BMI at inclusion (kg/m^2^)*	25 [23–28]	25 [22–27]	27 [24–30]
BMI at second exam (kg/m^2^)	25 [23–29]	25 [22–28]	27 [23–30]
Heart rate (bpm)	75 [65–90]	74 [65–90]	77 [70–92]
LVEF (%)*	30 ± 9	30 [25–36]	27 [22–31]
LVEDDi (mm/m^2^)	34 [31–38]	35 [32–39]	32 [30–37]
LVEDVi (mL/m^2^)*	115 [97–138]	120 [99–145]	98 [91–118]

ACE‐I, angiotensin‐converting enzyme inhibitor; ARB, angiotensin II receptor blocker; BMI, body mass index; CMP, cardiomyopathy; CRT, cardiac resynchronization therapy; HF, heart failure; ICD, internal cardioverter defibrillator; LVEDDi, left ventricular end‐diastolic diameter per m^2^; LVEDVi, left ventricular end‐diastolic volume per m^2^; LVEF, left ventricular ejection fraction; LVRR, left ventricular reverse remodelling.

* *P* < 0.05 in LVRR vs. non‐LVRR patients.

In *Table* 2, patients are divided into four groups according to BMI categories on admission: underweight (<18.5 kg/m^2^), normal weight (18.5–24.95 kg/m^2^), overweight (25–29.95 kg/m^2^), and obesity (>30 kg/m^2^). There was a significant increase in the rate of LVRR as well as an increase in the % of reduction in LVEDDi with increasing BMI.

**Table 2:** Clinical characteristics of patients, categorized according to body mass index classes

BMI class	<18.5 kg/m^2^ *n* = 13	18.5–24.9 kg/m^2^ *n* = 134	25–29.9 kg/m^2^ *n* = 101	≥30 kg/m^2^ *n* = 47
Age	54 [39–68]	57 [45–66]	59 [52–68]	60 [50–70]
Male gender	60%	83%	88%	79%
Diabetes	10%	27%	34%	38%
Hypertension	40%	58%	63%	70%
Ischaemic CMP	50%	46%	51%	47%
*De novo* HF	70%	44%	51%	47%
ACE‐I or ARB	100%	98%	96%	100%
Beta blockers	100%	90%	86%	94%
MRA	50%	54%	49%	55%
CRT	20%	15%	12%	13%
Heart rate (bpm)*	80 [72–92]	73 [65–88]	73 [63–87]	80 [70–97]
% change in BSA	0.4 [0–0.9]	0.1 [0–0.2]	0	0
% change in BMI	0.8 [−0.2 to 12]	0.1 [−0.1 to 0.2]	0.2 [−0.1 to 0.3]	0 [−0.1 to 0.1]
LVEDDi (mm/m^2^)	36 [36–43]	36 [34–39]	34 [31–37]	32 [30–34]
LVEDVi (mL/m^2^)	130 [93–157]	121 [99–149]	113 [95–131]	111 [92–134]
% change in LVEDDi*	1.4 [−4.3 to 8.8]	−1.2 [−7.2 to 4.5]	−1.9 [−11.9 to 4.0]	−3.8 [−12.7 to 2.9]
LVEF (%)	31 [20–40]	30 [25–35]	30 [25–35]	30 [25–37]
Change in LVEF (%)	0 [−10 to 5]	0 [−5 to 10]	2 [−1 to 6]	3 [−3 to 8]
LVRR**	0%	10%	26%	30%

ACE‐I, angiotensin‐converting enzyme inhibitor; ARB, angiotensin II receptor blocker; BMI, body mass index; BSA, body surface area; CMP, cardiomyopathy; CRT, cardiac resynchronization therapy; HF, heart failure; LVEDDi, left ventricular end‐diastolic diameter per m^2^; LVEDVi, left ventricular end‐diastolic volume per m^2^; LVEF, left ventricular ejection fraction; LVRR, left ventricular reverse remodelling; MRA, mineraloreceptor antagonists.

* *P* < 0.05 between BMI classes.

** *P* < 0.01 between BMI classes.

During a mean follow‐up of 37 ± 11 months, 32% of patients died (*n* = 85) or were transplanted (*n* = 9). Less MACE was observed in patients with LVRR than in patients without LVRR: 19% vs. 33% (OR 0.44, 95%CI 0.20–0.95, *P* = 0.03). Following variables were also associated with death or cardiac transplantation: *de novo* HF (*P* < 0.001), non‐use of angiotensin‐converting enzyme inhibitor or angiotensin II inhibitor use (*P* = 0.041), non‐use of cardiac resynchronization therapy use (*P* = 0.038), age (*P* = 0.015), and LVEF (*P* = 0.032). Relationship between BMI and death or transplantation was at limit of significance: OR 0.95 (95%CI 0.89–1.00), *P* = 0.072. In ensuing multivariate analysis, only presentation with *de novo* HF (OR 0.39, 95%CI 0.20–0.77, *P* = 0.006), age (OR 1.03, 95%CI 1.00–1.06, *P* = 0.039), and LVEF (OR 0.94, 95%CI 0.90–0.98, *P* = 0.005) remained predictors of death or cardiac transplantation during follow‐up.

## Discussion

Our study shows a significant relationship between BMI and LVRR in HF patients. This finding enlarges previous results about obesity paradox and points out a possible protective role of high BMI against LVR. In the field of chronic HF, high BMI has been associated with LVRR in a cohort of patient undergoing resynchronization therapy,^9^ but this association was not significant in other observational studies.^10^, ^11^ In our study, LVRR occurred in 18% of patients that is in line with previous studies using similar definition of LVRR.^7^, ^11^ As expected, LVRR occurred mainly in *de novo* HF patients and was negatively related to initial left ventricular dilation. Our study unravelled BMI as a predictor of LVRR, providing a pathophysiological underpinning of these earlier observations and directions for additional research in HF. Several recent observations might explain the protective effect of obesity in established HF. At first, the excess adipose tissue might act as an energy reservoir allowing the body to resist increased catabolic demands.^12^, ^13^ Secondly, the propensity of obese patients to develop arterial hypertension might make them more likely to tolerate target doses of evidence‐based HF therapies. Thirdly, obesity has been shown to impact circulating levels of neurohormones and cytokines related to HF pathogenesis.^14^ As examples, tumor necrosis factor alpha has been associated with cardiac cachexia and was inversely correlated with BMI^15^; increase in plasma adiponectin levels (related with increase in risk of mortality) are inversely correlated with BMI.^16^ High BMI might attenuate the release of detrimental circulating mediators once cardiovascular disease is present. Obesity had paradoxically been associated with a better prognosis in HF patients, referred to as the ‘obesity paradox’.^17^, ^18^ We could not identify BMI as an independent predictor of mortality, but there was a trend to a lower BMI in MACE patients, and LVRR was significantly less frequent in MACE patients. Recently, a U‐shaped curve was observed for short‐term prognosis according to BMI in a cohort of HF patients with various BMI including severe obesity.^19^ Our cohort included only very few patients with severe obesity, and thus, we cannot exclude such a U‐shaped curve.

In conclusion, our study provides further evidence for the existence of a protective effect of obesity in established cardiac disease, more specifically supporting the ‘obesity paradox’ in HF patients.

## Conflict of interest

None declared.
